# Stroma areactive invasion front areas (SARIFA) in 1,298 pT3/pT4 colorectal cancers: A strong prognostic parameter complementing established morphological criteria

**DOI:** 10.1111/his.15501

**Published:** 2025-07-04

**Authors:** Sebastian Foersch, Nuwar Harb, Anne‐Sophie Litmeyer, Wilfried Roth, Irina Breus, Johannes Schraml, Annika Weiß, Katja Steiger, Julia Teply‐Szymanski, Detlef K Bartsch, Carsten Denkert, Maxime Schmitt, Moritz Jesinghaus

**Affiliations:** ^1^ Institute of Pathology University Medical Center Mainz Germany; ^2^ Institute of Pathology Philipps‐University Marburg and University Hospital Marburg Marburg Germany; ^3^ Institute of Pathology Technical University of Munich Munich Germany; ^4^ Department of Surgery Philipps‐University Marburg and University Hospital Marburg Marburg Germany

## Abstract

**Aims:**

Colorectal carcinoma (CRC) is one of the most common cancers worldwide and is associated with significant morbidity and mortality. Histopathology plays a crucial role in the diagnosis, prognostication and treatment planning of CRC. In addition to well‐established morphological parameters such as tumour budding, grade and histopathological subtypes, the interaction between tumour cells and adipose tissue has gained increasing attention.

**Methods and results:**

The frequency of direct proximity between cancer cells and adipocytes, termed *Stroma Areactive Invasion Front Areas* (SARIFA), was assessed in a multicentre cohort of 1,298 patients with pT3/pT4 CRC. The prognostic impact of SARIFA was evaluated, with a particular focus on its relationship with established histopathological parameters. SARIFA was associated with adverse clinicopathological features and served as an indicator of poor prognosis across all survival metrics in the overall cohort (disease‐specific survival [DSS]; *P* < 0.001). This prognostic value remained significant across all pTNM stages (e.g. DSS in pT3, pN0, pN1/2, pM0 CRC; *P* < 0.001, respectively) and identified additional adverse prognostic subgroups within tumour budding and grade categories (DSS, *P* < 0.001, respectively) and within histopathological subtypes. In multivariable analyses, including pTNM stage and the aforementioned histopathological parameters, SARIFA remained a highly significant prognostic factor (DSS *P* < 0.001, hazard ratio: 1.73).

**Discussion:**

Our study confirms the high prognostic relevance of SARIFA across key clinicopathological subgroups of CRC and highlights its value as a meaningful complement to established morphological parameters. Given its ability to provide additional prognostic insights, our findings advocate for its inclusion in pathology reports to improve risk assessment and clinical management.

## Graphical abstract

SARIFA is a novel, highly prognostic, stage‐independent morphological parameter in CRC. It complements established histopathological prognostic markers and provides crucial additional prognostic information across key clinicopathological and morphological subgroups. Partially created in https://BioRender.com.

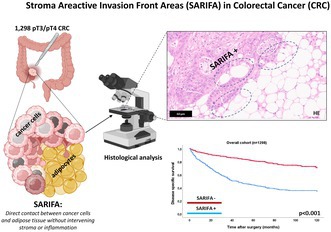

**Abbreviations**
Bd: tumour budding
CRC: colorectal carcinoma
DFS: disease‐free survival
DSS: disease‐specific survival
HE: Haematoxylin and Eosin
HR: hazard ratio
OS: overall survival
SARIFA: *Stroma Areactive Invasion Front Areas*
UICC: Union for International Cancer Control
WHO: World Health Organization

## Introduction

Colorectal carcinoma (CRC) is the third most common cancer worldwide in terms of both incidence and mortality.^1^, ^2^ Pathological pTNM staging, based on Union for International Cancer Control (UICC) guidelines, is the most widely accepted prognostic factor for CRC and generally serves as the foundation for post‐operative clinical decision‐making.^3^, ^4^ Nonetheless, a substantial number of cases exhibit a disease course that does not align with their pTNM stage, underscoring the clinical need for additional prognostic parameters beyond pTNM staging. Morphologically, CRC demonstrates an exceptional degree of heterogeneity, characterized by a wide array of diverse architectural and cellular features. Consequently, additional histological parameters that assess the tumour's composition from various perspectives are routinely included in pathological reports to provide supplementary prognostic information.^5^, ^6^, ^7^

In its current 5th edition, the WHO classification for colorectal carcinoma (CRC) includes three ‘essential and desirable’ morphological criteria regarding the tumour architecture, based on the evaluation of Haematoxylin and Eosin (HE)‐stained slides,^5^, ^6^ which are recommended for inclusion in pathological reports. These include tumour budding, defined as invasive complexes of fewer than five cancerous cells, which serves as a marker of the tumour's capacity for dissociative growth and is categorized into three grades (Bd1, Bd2, Bd3) according to international consensus guidelines^8^, ^9^; tumour grade (low vs. high grade), which assesses the extent of glandular formation^10^; and identification of the histopathological subtype. Previous studies have confirmed the general prognostic significance of these factors.^5^, ^7^, ^8^, ^9^, ^11^, ^12^, ^13^, ^14^

The presence or absence of so‐called *Stroma Areactive Invasion Front Areas* (SARIFA) has recently been proposed as a promising addition to the portfolio of purely HE‐based morphological biomarkers. SARIFA is defined as direct tumour‐adipocyte adjacency at the tumour invasion front, where a group of at least ≥5 tumour cells is in direct contact with adipocytes, without an intervening desmoplastic stromal or inflammatory reaction. Previous studies have associated SARIFA‐positivity with a strikingly worse prognosis in various epithelial cancers (e.g. gastric, pancreatic, prostate, colorectal) and also highlighted, that SARIFA assessment is straightforward with low interobserver variability.^15^, ^16^, ^17^, ^18^, ^19^, ^20^, ^21^

However, for CRC, some open questions remain: Previous studies have confirmed the natural assumption that the SARIFA phenomenon almost exclusively occurs in locally advanced cancers that have at least invaded the circumferential adipose tissue (pT3/pT4).^19^, ^22^ Therefore, it remains to be determined how SARIFA affects patient prognosis when only these locally advanced tumours are considered, where SARIFA is most likely to occur. Furthermore, it is still unclear how SARIFA is related to the already established morphological criteria of CRC given by the WHO classification (tumour budding, tumour grade, Histopathological subtypes) and how its prognostic performance relates in comparison to these already established parameters.

In order to address these questions, we assessed the SARIFA status in a very large multicentre cohort of 1,298 patients with pT3/pT4 colorectal cancer (CRC) and correlated the results with patient survival, emphasizing its relationship and prognostic performance compared to other morphological parameters.

## Patients and Methods

### Multicentric CRC Cohort

Our main study cohort comprised 1,298 patients with pT3/pT4 CRC who underwent surgical resection between 1997 and 2019 at three tertiary care centres in Germany: University Hospital Klinikum rechts der Isar, Munich; University Hospital Mainz and University Hospital Marburg. Of the 1,298 patients, 722 (55.6%) were male, with a median age of 71 years. Right‐sided (611/1,298; 47.1%) and left‐sided CRCs (687/52.9%) were nearly equally distributed. Postoperative pathological staging was based on the eighth edition of the TNM classification of malignant tumours, identifying 591 patients (45.5%) in UICC stage II, 489 (37.7%) in stage III and 218 (16.8%) in stage IV. Survival data were collected from local cancer registries and/or hospital records. Endpoints for all survival analyses were defined as either events or loss to follow‐up, in which case patients were censored at the date of their last recorded entry. Patients without events within 120 months were censored at that timepoint. The detailed clinicopathological features of the cohort are given in Supplementary Table 1.

### Histopathologic Evaluation

All available full‐block Haematoxylin and Eosin‐stained slides from the resection specimens of 1,298 patients were reviewed by an experienced gastrointestinal pathologist (MJ) using an Olympus BX46 microscope. Of these, 911 cases (70.2%) had two or more evaluable slides, whereas 387 cases (29.8%) had only one. For each case, all available tumour‐bearing slides were included in the analysis.

### Assessment of SARIFA‐Status

**Figure 1:**
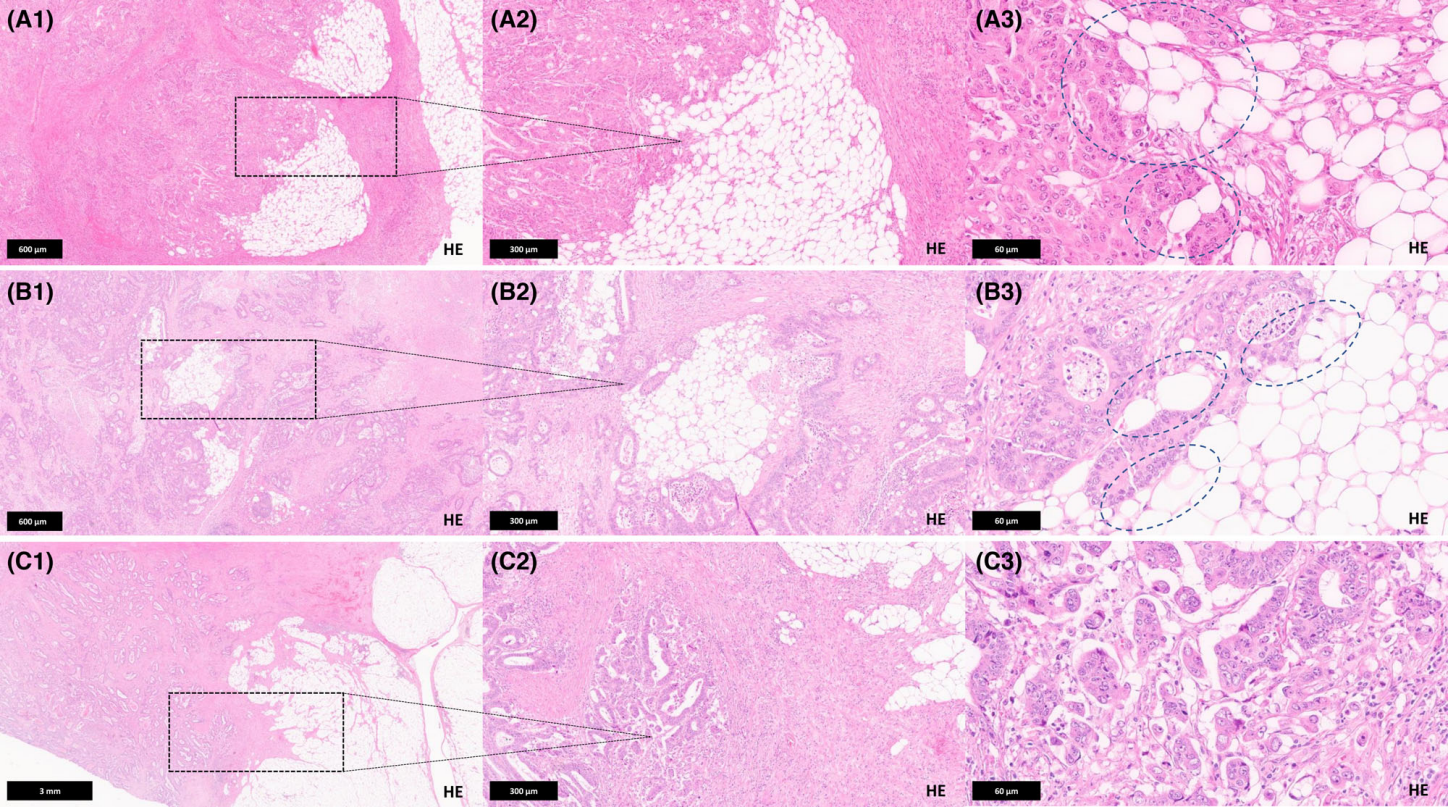
Introduction of SARIFA as a histopathological parameter in colorectal cancer. (**A** and **B**) Examples of an SARIFA‐positive CRCs. (**A1**) Shows a low magnification (4×) illustrating the invasion of pericolic fat tissue. (**A2**) Shows an intermediate magnification (10×) at the invasive front where tumour cells are closely associated with adipocytes. (**A3**) Shows a high magnification area with several spots (circles) where groups of more than five tumour cells are adjacent to adipocytes without an intervening desmoplastic stromal reaction or inflammatory infiltrate. (**B1**) shows a low magnification (4×) illustrating the invasion of pericolic fat tissue, with fibrotic areas as well as regions where adipocytes are still visible. (**B2**) Shows an intermediate magnification (10×) at the invasive front where tumour cells are closely associated with remaining adipocytes. (**B3**) Shows a high magnification area with several spots (circles) where groups of more than five tumour cells are adjacent to adipocytes without an intervening desmoplastic stromal reaction or tumour‐associated inflammation. (**C**) Example of a SARIFA‐negative CRC. (**C1**) Shows a scanning magnification (1.25×) of a CRC with broad invasion of the pericolic fat. (**C2**) Shows the invasive tumour accompanied by a dense desmoplastic reaction. At intermediate magnification (10×), it is already apparent that no adipocytes are present adjacent to the tumour cells. (**C3**) Shows tumour cells at high magnification (40×), embedded in a dense desmoplastic reaction. The slit‐like, optically clear spaces surrounding the cancer cells represent retraction artefacts, which must be clearly distinguished from SARIFA, as no direct contact with adipocytes is present.

SARIFA was defined as the presence of direct tumour‐adipocyte adjacency at the tumour invasion front, characterized by at least one cluster of ≥5 tumour cells in direct contact with adipocytes, without an intervening desmoplastic stromal or inflammatory reaction. A single area exhibiting this feature was sufficient to classify a CRC as SARIFA‐positive. Cases without any detectable areas of direct tumour‐adipocyte adjacency were classified as SARIFA‐negative. Histopathological examples of SARIFA‐positive and negative CRC are given in Figure 1.

### Assessment of Tumour Budding, Tumour Grade and Histopathological CRC Subtype

All tumours from the main cohort were also analysed for the distribution and prevalence of the morphology‐based ‘essential and desirable criteria’ defined in the 2019 WHO classification (tumour budding, tumour grade, histological subtype). Most neoplasms in our cohort had previously been characterized regarding these parameters as part of earlier studies^7^, ^23^, ^24^; additional tumours recruited for this study were reclassified as previously described. Briefly, tumour budding was defined as the presence of single cells or clusters of fewer than five cells at the invasive front and assessed on H&E‐stained slides using a three‐tiered scoring system [Bd1 (low budding activity): 0–4 buds, Bd2 (intermediate budding activity): 5–9 buds, Bd3 (high budding activity): >10 buds per 0.785 mm^2^ at 20× magnification], following the international consensus guidelines. All cases were also classified according to the current WHO colorectal carcinoma subtypes. Tumour grade was determined based on gland formation, subdividing CRCs into ‘low grade’ (≥50% gland formation) and ‘high grade’ (<50% gland formation). Histopathological examples of the aforementioned parameters are given in Figure S1.

### Statistical Analyses

Statistical analyses were performed using SPSS version 28 (SPSS Institute, Chicago, IL). Chi‐squared tests, Fisher's exact test and the Bonferroni method were applied as appropriate. Survival analyses included the Kaplan–Meier method with log‐rank test, univariate Cox regression and multivariate analysis using the Cox proportional hazards model. All tests were two‐sided, and p‐values ≤0.05 were considered statistically significant.

## Results

### Prognostic Impact of pTNM‐Staging, Tumour Budding, CRC Subtypes and Tumour Grade

As expected, pT, pN, pM, UICC stage and resection status significantly impacted OS, DSS and DFS (*P* < 0.001 for all) in univariable survival analyses. Tumour budding strongly influenced all survival parameters (*P* < 0.001), with markedly reduced survival in high‐budding patients (Bd2/Bd3 vs. Bd1; Hazard Ratio (HR, univariable) for DSS: Bd2: 4.1, Bd3: 9.0). Histopathological CRC subtypes also had a significant impact (*P* < 0.001; Table 1): adenoma‐like (HR, univariable: 0.13) and medullary adenocarcinoma (HR, univariable: 0.06) were linked to prolonged survival, while micropapillary adenocarcinoma (HR, univariable: 2.25) and neuroendocrine carcinomas (HR, univariable: 6.68) indicated poor prognosis. Tumour grade significantly affected survival (*P* < 0.001; Table 1, HR, univariable for DSS: 1.90). For details, see Table S1.

### Prevalence of SARIFA and its Association with Clinicopathological and Central Histopathological Parameters of CRC

**Figure 2:**
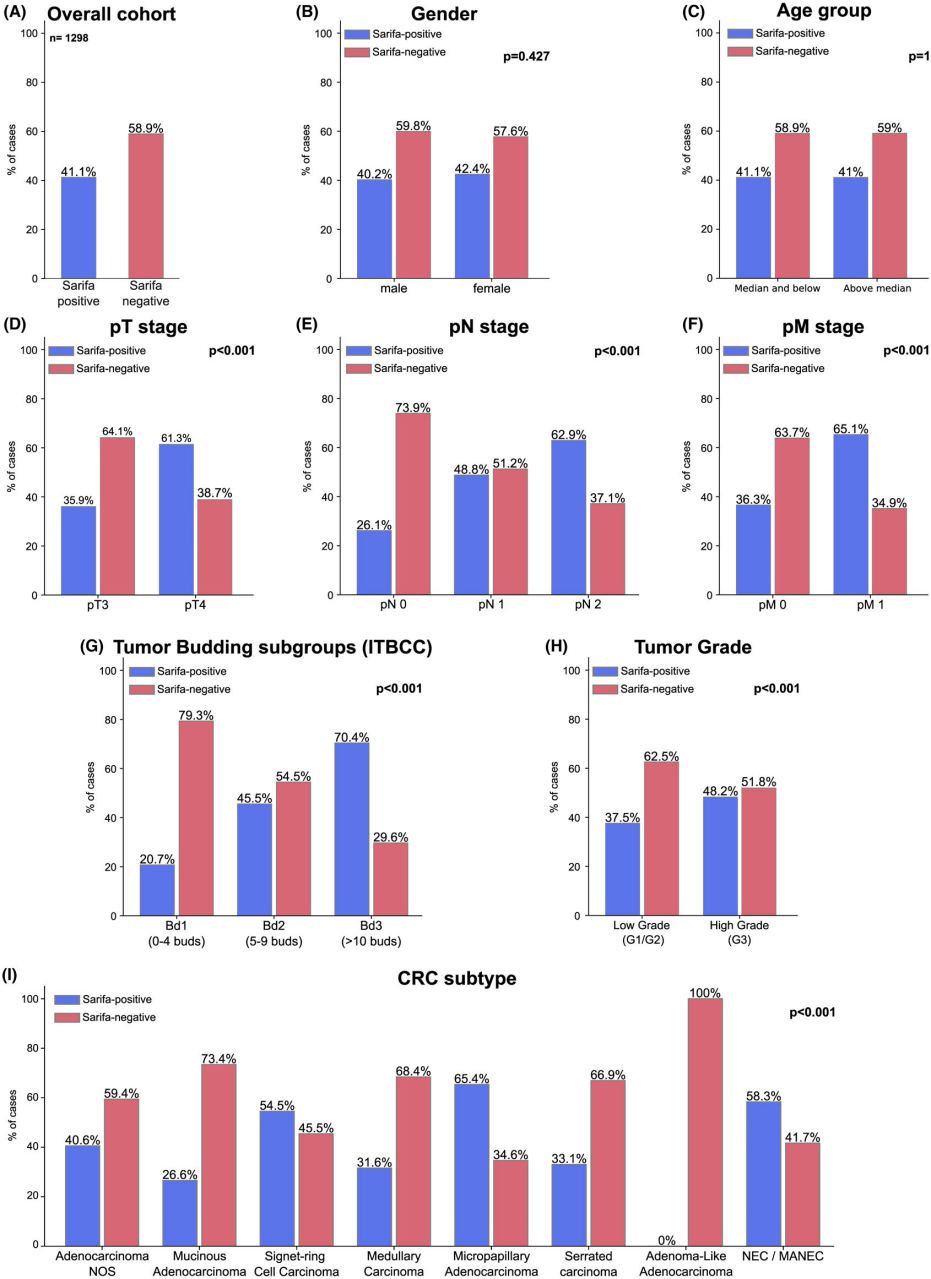
Frequency and association of SARIFA status with clinicopathological features and morphological parameters. (A‐I) Frequency and association of SARIFA status with clinicopathological features and morphological parameters.

As depicted in Figure 2, 534 CRC cases (41.1%) were SARIFA‐positive and 764 (58.9%) SARIFA‐negative. SARIFA‐positivity was strongly linked to adverse clinicopathological features, being more frequent in pT4 CRC, those with regional lymph node metastases (pN1/2 vs. pN0), distant metastases (pM0 vs. pM1) and positive resection margins (R0 vs. R1/2) (each *P* < 0.001). SARIFA‐positivity correlated with adverse histopathological features, being more prevalent in high‐grade CRC, increased tumour budding (Bd2/Bd3), and histological subtypes associated with advanced stage and other adverse histological features (e.g., micropapillary adenocarcinoma, neuroendocrine carcinoma) (each *P* < 0.001). No significant association was found with gender, age or tumour location. Inter‐observer variability of SARIFA was probed on 150 randomly selected cases with investigation of a second histopathologist (M.S.) and demonstrated an excellent interobserver agreement (*P* < 0.001, Cohen's kappa coefficient: 0.917). The discrepant cases were discussed on a discussion microscope until a consensus was reached.

### Prognostic Impact of SARIFA in the Overall Cohort and in Clinicopathological Subgroups

**Figure 3:**
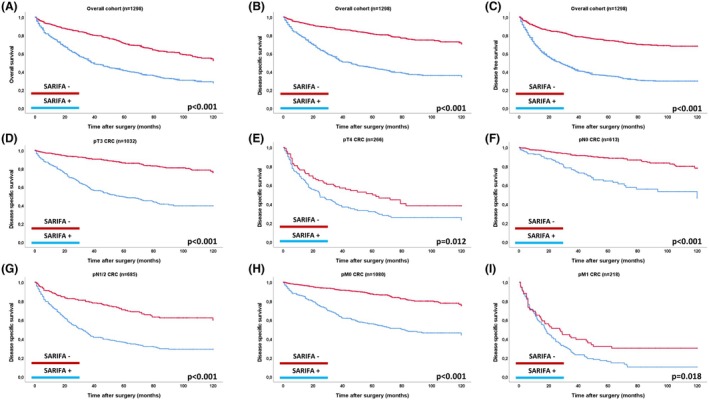
Prognostic impact (univariable) of SARIFA in the overall cohort on overall‐, disease‐specific and disease‐free survival (A–C) and prognostic impact on disease specific survival across pTNM subgroups (D–I). [Colour figure can be viewed at wileyonlinelibrary.com]

In the overall cohort of all 1,298 CRC, SARIFA‐positive cases were associated with a significantly poorer prognosis across all survival metrics in univariable analyses (OS, DSS, DFS: *P* < 0.001 for all; see Table S1, Figure 3). For example, the mean DSS for SARIFA‐positive cases was 60.72 months, compared to 99.04 months for SARIFA‐negative CRCs [Hazard Ratio (HR) for DSS for SARIFA‐positive CRC: 3.72]. The strong prognostic impact of SARIFA remained consistent in separate subgroup analyses, including pTNM categories (pT3 vs. pT4, pN0 vs. pN1/2, pM0 vs. pM1, Figure 3), tumour locations (right‐ vs. left‐sided CRC), men versus women and between age groups (Figure S2).

### Prognostic Impact of SARIFA across Subgroups of Tumour Budding, Tumour Grade and within Histopathological Subtypes

**Figure 4:**
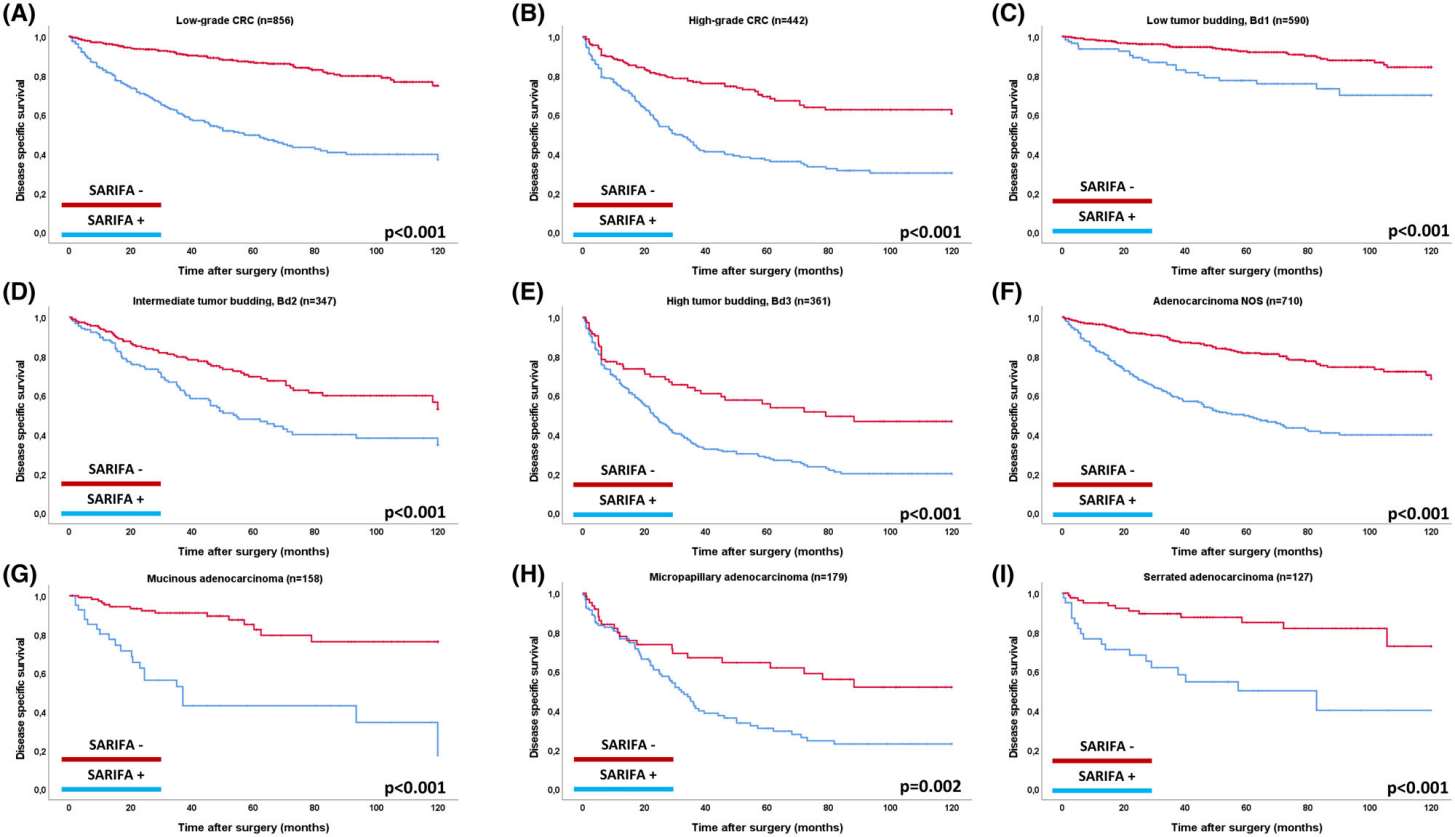
Prognostic impact (univariable) of SARIFA on disease‐specific survival across key morphological parameters of CRC. (A–I) Prognostic impact (univariable) of SARIFA on disease‐specific survival across key morphological parameters of CRC. [Colour figure can be viewed at wileyonlinelibrary.com]

As shown in Figure 4, SARIFA retained strong prognostic value across key histopathological subgroups. SARIFA‐positive cases had a significantly worse prognosis in both low‐grade (DSS, *P* < 0.001; HR, univariable for DSS: 4.48) and high‐grade CRC (*P* < 0.001; HR, univariable for DSS: 2.62). It also identified prognostic subgroups within tumour budding categories, showing worse outcomes in SARIFA‐positive CRC with low (*P* < 0.001; HR for DSS: 2.79), intermediate (*P* < 0.001; HR: 1.89) and high budding activity (*P* < 0.001; HR: 1.99). SARIFA had a strong prognostic impact in the most common histopathological CRC subtypes. In adenocarcinoma NOS, SARIFA‐positive cases had significantly poorer outcomes (DSS, *P* < 0.001; HR, univariable for DSS: 3.36), as did cases with mucinous (*P* < 0.001; HR, univariable: 5.72), micropapillary (DSS *P* = 0.002, HR, univariable: 2.10) and serrated adenocarcinoma (*P* < 0.001; HR, univariable: 4.08).

### Multivariable Analyses

As depicted for DSS in Table 1, SARIFA also maintained its prognostic relevance in multivariable statistical analyses including pTNM‐staging, tumour localization, resection status, gender, age as well as tumour budding, tumour grade and histopathological subtypes. SARIFA maintained its strong prognostic relevance in all multivariable survival comparisons (DSS *P* < 0.001, HR multivariable: 1.73), which was also the case for tumour budding and histopathological subtypes (DSS, *P* < 0.001 for all; HR for DSS see Table 1). Tumour grade showed a lower but still significant prognostic demarcation for DSS (*P* = 0.014, HR multivariable = 1.31).

**Table 1:** Multivariable analysis of SARIFA on disease‐specific survival including pTNM staging, resection status, tumour localization, other histopathological parameters, age and gender

	HR (DSS)	Lower CI (95%)	Upper CI (95%)	*P*‐value
SARIFA status				<0.001
SARIFA negative	1.00			
SARIFA positive	1.73	1.37	2.19	
Tumour budding activity				<0.001
Bd1 (no/low tumour budding)	1.00			
Bd2 (intermediate tumour budding)	2.48	1.79	3.44	
Bd3 (high tumour budding)	4.08	2.89	5.77	
Histopathological subtypes				<0.001
Adenocarcinoma NOS	1.00			
Mucinous Adenocarcinoma	0.95	0.66	1.38	
Signet‐ring cell Carcinoma	1.14	0.46	2.83	
Medullary Carcinoma	0.07	0.01	0.52	
Micropapillary Adenocarcinoma	0.76	0.59	1.02	
Serrated Carcinoma	1.09	0.74	1.60	
Adenoma‐like Adenocarcinoma	0.48	0.07	3.50	
NEC/MANEC	2.28	1.41	3.70	
pT				<0.001
pT3	1.00			
pT4	1.88	1.51	2.35	
pN				<0.001
pN0	1.00			
pN1	1.37	1.04	1.78	
pN2	2.07	1.68	2.72	
pM				<0.001
pM0	1.00			
pM1	2.14	1.68	2.72	
Resection status				0.28
R0	1.00			
R1	1.21	0.86	1.69	
R2	1.11	0.71	1.74	
WHO grade				0.02
Low grade	1.00			
High grade	1.31	1.04	1.63	
Gender				0.13
Male	1.00			
Female	0.85	0.69	1.05	
Age				<0.001
Below median	1.00			
Median and above	1.68	1.36	2.06	
Tumour localization				0.96
Right‐sided (coecum/ascendens/transversum)	1.00			
Left‐sided (descendens/sigmoideum/rectum)	1.01	0.82	1.23	

### Impact of Sampling on SARIFA Detection

In pT3 tumours, SARIFA was significantly more frequently detected when two or more slides were available compared to those with only one available slide (38.2% vs. 31.3%, *P* = 0.049), indicating that sampling may indeed influence the detection rate in this subgroup. When analysing disease‐specific survival (DSS) separately for pT3 cases with one slide and those with two or more, SARIFA remained a highly significant prognostic factor in both subgroups (*P* < 0.001; HR in pT3 CRC with ≥2 available slides: 4.19; HR in pT3 CRC with one available slide: 5.52). In cases with only one slide, mean DSS was 105.5 months for SARIFA‐negative tumours versus 61.3 months for SARIFA‐positive tumours; in cases with ≥2 slides, mean DSS was 103.9 months versus 65.8 months, respectively (data not shown).

In pT4 tumours, the number of available slides did not significantly affect SARIFA detection. In this subgroup, the vast majority of cases (91.4%) included in our cohort had two or more evaluable slides, and only a small proportion of cases were based on a single section showing tumour invasion of pericolic or perirectal adipose tissue. Due to the low number of pT4 cases with only one slide, the prognostic relevance of SARIFA was preserved only in cases with two or more slides (*P* = 0.024, HR = 1.52).

## Discussion

Colorectal carcinoma (CRC) encompasses a wide range of morphological features. According to the current WHO classification, three essential morphology‐based features—tumour budding, tumour grade and histological subtype—should be included in pathology reports.^5^, ^6^ Recent studies across various carcinoma types have introduced Stroma Areactive Invasion Front Areas (SARIFA) as a novel morphology‐based feature with significant prognostic relevance.^15^, ^16^, ^17^, ^18^, ^19^, ^20^, ^21^, ^22^, ^25^

SARIFA describes the direct interaction between tumour cells and adipocytes, defined by a at least one cluster of at least five tumour cells in direct contact with non‐neoplastic fat cells, without intervening desmoplastic stroma or inflammation. The most extensive studies to date have focused on patients with gastric adenocarcinoma, showing that SARIFA‐positive cases are associated with significantly poorer survival and adverse clinicopathological characteristics. Similar findings have been noted in smaller studies of pancreatic ductal adenocarcinoma and prostatic carcinoma.^17^

In CRC, prior research involving mixed cohorts across all pT stages has demonstrated that SARIFA is a highly prognostic feature. However, these studies have also shown that SARIFA is most commonly observed in locally advanced CRCs (pT3/pT4) and is rarely seen in lower pT‐stage (pT1/pT2) tumours.^18^, ^19^ This observation prompted us to investigate the prognostic significance of SARIFA specifically within pT3/pT4 CRC, where this phenomenon is more prevalent. Additionally, understanding the relationship between SARIFA and established key morphological parameters, as well as evaluating SARIFA's prognostic performance in comparison to these parameters, remained largely unexplored and was another key aim of our study.

Our analysis of a very large cohort of 1,298 pT3/pT4 CRC cases independently validated SARIFA‐positivity as a poor prognostic factor within the subset of CRC where this phenomenon is observed, providing compelling evidence for its inclusion in CRC pathology reports. Notably, this strong prognostic effect was maintained within separate analyses of pT3 and pT4 CRC and also observed within both nodal‐negative (pN0) and nodal‐positive patients (pN1 and pN2) as well in patients with and without distant metastasis. Furthermore, our study underscores strong associations between SARIFA and the morphology‐based features designated as ‘essential and desirable’ in the current WHO classification. SARIFA‐positive tumours were significantly more prevalent in cases with increased tumour budding activity and high‐grade tumours or CRC subtypes associated with advanced stage and other adverse histological factors, while being less frequent in low‐risk groups, indicating an intrinsic relationship between these factors. Notably, our findings showed that SARIFA could distinguish distinct prognostic subgroups even within low‐ and high‐risk categories of these parameters, adding an even deeper level of prognostic stratification. In multivariable analyses that included pTNM staging and the aforementioned established histopathological parameters, SARIFA retained this strong prognostic relevance across all survival metrics and outperformed tumour grade. These findings emphasize the robust prognostic capability of SARIFA and support its integration into routine histological assessments of locally advanced CRC to improve patient risk stratification and guide clinical decision‐making. It should be noted that only unequivocal foci of SARIFA—defined by direct contact between tumour cells and clearly identifiable adipocytes—should be diagnosed as such in routine pathology. Observers should remain mindful of potential morphological mimickers, such as retraction artefacts or dense histiocytic infiltrates, which may resemble SARIFA. Nonetheless, these potential mimickers can generally be reliably distinguished from true SARIFA, as indicated by the previously reported low interobserver variability,^26^ which was also confirmed by exploratory interobserver testing in the present study.

Another notable finding of our study is that the detection of SARIFA appears to be influenced by the extent of histological sampling. Tumours with two or more evaluable slides showing invasion into pericolic or perirectal adipose tissue had a significantly higher likelihood of being classified as SARIFA‐positive. Although the prognostic relevance of SARIFA remained robust regardless of the number of slides, all available tumour‐bearing sections should be carefully assessed in routine diagnostic pathology before confidently classifying a case as SARIFA‐negative.

Besides its retrospective nature, one major limitation of our study is that in approximately one third of cases, only a single diagnostic slide was available for SARIFA assessment. This may have led to an underestimation of SARIFA‐positive tumours. Nevertheless, the strong prognostic relevance of SARIFA was preserved regardless of the number of evaluated sections. In addition, the prognostic impact of the extent or multifocality of SARIFA was not systematically assessed in this study. Future investigations—potentially supported by digital image analysis—are needed to determine whether the number or distribution of SARIFA foci carries additional prognostic significance. Moreover, while our study provides strong histomorphological evidence for the prognostic relevance of SARIFA, it does not investigate the underlying biological mechanisms. Although previous studies suggest that SARIFA is not driven by specific genetic alterations but rather associated with dysregulated lipid metabolism,^17^ further research is needed to better elucidate its molecular basis.

In conclusion, our study validates SARIFA as a strong and independent prognostic factor in pT3/pT4 colorectal cancer, underscoring its potential as an important addition to established histopathological criteria. Given its ability to further stratify risk within key prognostic subgroups, SARIFA should be integrated into pathology reports of CRC to enhance patient risk assessment and clinical decision‐making. Future prospective studies should investigate its potential implications for treatment strategies and personalized therapeutic approaches.

## Author Contributions

MJ and SF designed this study. MJ and SF wrote the manuscript with assistance from CD, ASL, MS and DKB. MJ, KS, SF, IB, JS, WR and CD performed histopathological analysis. MJ, NH, AW, JTS and SF performed statistical analyses. MJ, DKB, SF, WR, JTS, IB and JS collected clinicopathological data.

## Funding

Funded by a grant of the Deutsche Krebshilfe (German Cancer Aid): TargHet (‘Targeting Inter‐ and Intratumoral Heterogeneity in Colorectal Cancer: Integration of Artificial Intelligence, Spatial Sequencing and Patient‐Derived Organoids’, Project‐ID 70115995) to SF, CD and MJ. The authors disclose that they have no significant relationships with, or financial interest in, any commercial companies pertaining to this article.

## Conflicts of Interest

The authors declare no conflicts of interest. Funded by a grant of the Deutsche Krebshilfe (German Cancer Aid): Project‐ID 360372040—SFB 1335 to SF, CD and MJ.

## Supporting information


**Figure S1.** Examples of the essential histopathological criteria defined by the 2019 WHO classification of digestive tumors.

**Figure S2.** Prognostic impact (univariable analysis) on disease‐specific survival within subgroups.

**Table S1.** Clinicopathological characteristics of the cohort and their association with survival parameters.

## Data Availability

The data that support the findings of this study are available on request from the corresponding author. The data are not publicly available due to privacy or ethical restrictions.
